# The Genus *Cerion* (Gastropoda: Cerionidae) in the Florida Keys

**DOI:** 10.1371/journal.pone.0137325

**Published:** 2015-09-17

**Authors:** Yesha Shrestha, Herman H. Wirshing, M. G. Harasewych

**Affiliations:** 1 Department of Biochemistry and Molecular Medicine, School of Medicine and Health Sciences, George Washington University, Washington, D.C., United States of America; 2 Department of Invertebrate Zoology, National Museum of Natural History, Smithsonian Institution, Washington, D.C., United States of America; University of California, UNITED STATES

## Abstract

The systematic relationships and phylogeography of *Cerion incanum*, the only species of *Cerion* native to the Florida Keys, are reviewed based on partial sequences of the mitochondrial COI and 16S genes derived from 18 populations spanning the range of this species and including the type localities of all four described subspecies. Our samples included specimens of *Cerion casablancae*, a species introduced to Indian Key in 1912, and a population of *C*. *incanum* x *C*. *casablancae* hybrids descended from a population of *C*. *casablancae* introduced onto Bahia Honda Key in the same year. Molecular data did not support the partition of *C*. *incanum* into subspecies, nor could populations be apportioned reliably into subspecies based on morphological features used to define the subspecies. Phylogenetic analyses affirmed the derived relationship of *C*. *incanum* relative to other cerionids, and indicated a Bahamian origin for the *Cerion* fauna of southern Florida. Relationships among the populations throughout the Keys indicate that the northernmost populations, closest to the Tomeu paleoislands that had been inhabited by *Cerion petuchi* during the Calabrian Pleistocene, are the oldest. The range of *Cerion incanum* expanded as the archipelago that is the Florida Keys was formed since the lower Tarantian Pleistocene by extension from the northeast to the southwest, with new islands populated as they were formed. The faunas of the High Coral Keys in the northeast and the Oölite Keys in the southwest, both with large islands that host multiple discontinuous populations of *Cerion*, are each composed of well supported clades that are characterized by distinctive haplotypes. In contrast, the fauna of the intervening Low Coral Keys consist of a heterogeneous series of populations, some with haplotypes derived from the High Coral Keys, others from the Oölite Keys. Individuals from the *C*. *incanum* x *C*. *casablancae* hybrid population inhabiting the southeastern coast of Bahia Honda Key were readily segregated based on their mitogenome lineage, grouping either with *C*. *incanum* or with *C*. *casablancae* from Indian Key. Hybrids with *C*. *casablancae* mitogenomes had haplotypes that were more divergent from their parent mitogenome than were hybrids with *C*. *incanum* mitogenomes.

## Introduction

The land snail family Cerionidae inhabits islands of the tropical western Atlantic, living on near shore terrestrial vegetation from southern Florida throughout the Bahamas, Greater Antilles, Cayman Islands, western Virgin Islands, and Dutch Antilles, but does not occur in Jamaica, the Lesser Antilles or coastal Central and South America. It is well known for its exceptional morphological diversity in shell form, which has given rise to nearly 500 species level taxa in the Recent fauna [1,2,3]. Variation in shell form within populations is generally low, while different populations, some separated by less than 200 m, may be distinguished by multiple, distinctive morphological characters [2], yet capable of interbreeding and hybridization [2,4]. Cerionids have served as models for the study of a variety of biological questions, among them relationships between morphological and genetic variation [5,6,7,8], dynamics of hybrid zones [9,10], and persistence of hybrids through time [11,12].

A review of the diversity within Cerionidae from geographic and temporal perspectives has shown it to be concentrated in the Pleistocene to Recent faunas of the Bahamas and Cuba [13]. It has been suggested that morphological diversity was amplified in these archipelagoes by repeated recombination (hybridization) and isolation of populations of neighboring islands that were conjoined during glaciations and separated during interglacial periods [13].

In contrast to the Bahamas and Cuba, the cerionid fauna of Florida is older and significantly less diverse. Two species (one with two subspecies) are known from the Late Oligocene-Early Miocene Ballast Point Silex Beds near Tampa, and a single species inhabited the Tomeu Paleoislands in what is now Loxahatchee, Palm Beach County, during the Calabrian Pleistocene [13]. Although only a single Recent species (subdivided into four subspecies on the basis of shell morphology) is native to Florida and confined to the Florida Keys, thirteen non-native cerionid species from throughout the Bahamas, Cuba, Puerto Rico and Curaçao had been introduced intentionally into the Florida Keys and Dry Tortugas between 1912 and 1924 as part of a well-documented series of studies [14,15]. Another large colony of *Cerion* with distinctive shell morphology occurs along the coastline in the region around Boynton Beach, Florida. These snails have been shown to be hybrids of at least two of five Cuban species introduced to Fort Jefferson in the Dry Tortugas in 1924. While this form no longer survives in the Dry Tortugas, a sample intentionally introduced from Ft. Jefferson onto a private estate in Boynton Beach in the late 1940's gave rise to the current, thriving population [16].

The Florida Keys were formed since the lower Tarantian Pleistocene [<132,000 years before present (YBP)], when sea levels were about 8 meters higher than they are today. As sea levels were lowered during the Wisconsin glaciation (~ 100,000 YBP), the coral reefs that had developed in the upper Keys were exposed and eroded. Oölite formed biogenetically and accumulated in the lower Keys. The Keys archipelago consists of four main lithological sections. The Key Largo Formation is older, and composed of exposed, uplifted coral limestone. It has been divided into the High Coral Keys (Key Biscayne to Plantation Key) and the Low Coral Keys (Windley Key to Bahia Honda Key). These islands are narrow and form a curved arc. The Miami Formation is younger and composed of oölitic limestone deposited in shallow water. The southern shorelines of these Oölite Keys (Big Pine Key westward to Key West and Boca Grande) continue along the arc formed by the High and Low Coral Keys, but the Oölite Keys tend to be elongated perpendicular to this arc. The Distal Atolls (Marquesas and Dry Tortugas) are Late Pleistocene and Holocene features mostly composed of living coral reefs [17].

The species *Cerion incanum* (Leidy, 1851) [18] is endemic to the Florida Keys, ranging from Key Biscayne to Key West, inhabiting the High Coral, Low Coral and Oölite Keys, but not the Distal Keys. This species has a complex nomenclatural history, with the name first introduced based on anatomical studies [18, 19]. Its type locality was subsequently restricted to the area around the salt ponds on Key West [20], and a neotype designated from this locality [19]. The name *Cerion incanum fasciata* (Binney, 1859) was proposed for an illustrated variety from Key Biscayne, Florida with irregular brownish longitudinal strips on a brownish base color [21]. Another phenotype with a somewhat larger shell with a tapering spire and frequently costate last whorl was given the name *Cerion incanum saccharimeta* by Pilsbry and Vanatta, 1899 [22], based on specimens from Sugarloaf Key. An additional thin-shelled form with a thin, narrow peristome and livid color from "Key Vaccas" was designated *Cerion incanum* var. *vaccinum* Pilsbry, 1902[20]. All of these form names have subspecific rank (ICZN Article 45.6.4)[23].

Early authors [24, 25, 26] considered *Cerion incanum* to be of Cuban origin, transported to the Florida Keys on floating debris and subsequently dispersing throughout the archipelago from southwest to northeast. Later authors [27] suggested that the *Cerion* of the Florida Keys were of Bahamian origin. The recent discovery of the *Cerion petuchi* Harasewych, 2012 [13] from the Bermont Formation (~1.6 M YBP) of Palm Beach County, a species most closely related to *C*. *agassizii* Dall, 1894, from Pleistocene deposits of the Great Bahama Bank, indicates Bahamian affinities for the ancestor of *Cerion incanum*. A phylogeny of Cerionidae based on partial COI sequences [16] indicates a well-supported relationship between *C*. *incanum* and species of *Cerion* from Andros Island and Eleuthera, both on the Great Bahama Bank. This suggests that the populations of the High Coral Keys are the oldest, and have the oldest genotype. As the archipelago that is the Florida Keys was formed during the Pleistocene by extension from the northeast to the southwest, the range of *Cerion incanum* expanded in a southwesterly direction, populating the new islands as they were formed. The genetic relatedness among the populations of the various keys is expected to reflect this pattern.

In the present study, we use partial sequences of the mitochondrial cytochrome c oxidase I (COI) and 16S rDNA genes to investigate genetic variation among and within populations of *Cerion* inhabiting the Florida Keys in order to infer the relationships among them, and to gain insights into biogeographic patterns within the Keys as well as the interactions between native and introduced species.

## Materials and Methods

### Sample Collection

**Fig 1:**
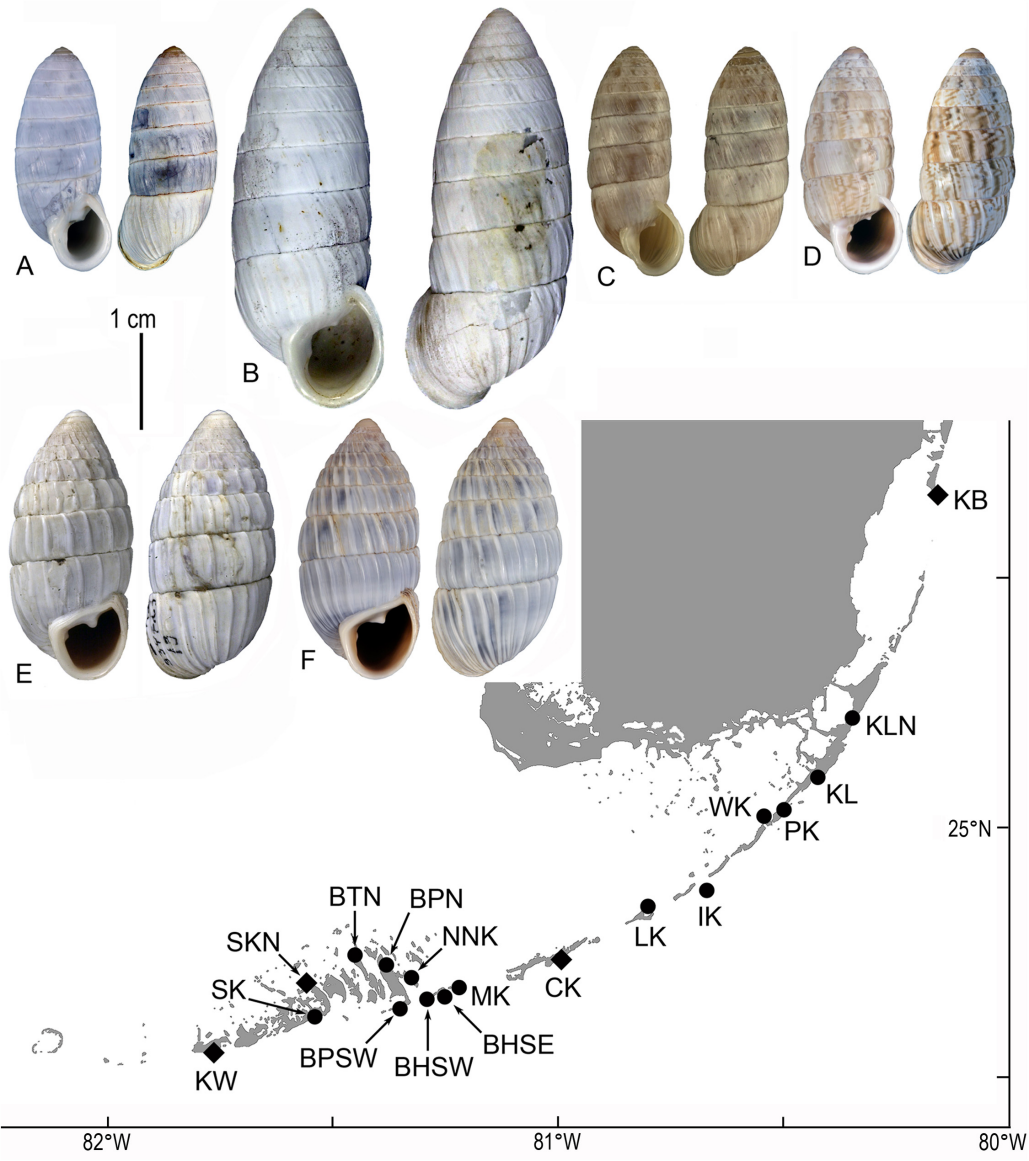
Locations of sampled populations and exemplars of phenotypes of *Cerion* encountered in this study: A. *Cerion incanum incanum* (Leidy, 1851), neotype, USNM 1231355, Key West. B. *Cerion incanum saccharimeta* Pilsbry & Vanatta, 1899, lectotype, ANSP 73483, Sugar Loaf Key. C. *Cerion incanum vaccinum* Pilsbry, 1902, lectotype, ANSP 67964, Key Vaccas. D. *Cerion incanum fasciata* (Binney, 1859), USNM 1175006, Key Biscayne. E-F. *Cerion casablancae* Bartsch, 1920. E. Lectotype, USNM 334723, Andros Island, Bahamas, collected 1912. F. USNM 1175018, Indian Key, collected 2012. Locations of sampled populations (solid circles) are identified by station letter designations (Table 1) Diamonds correspond to type localities for the named subspecies.

**Fig 2:**
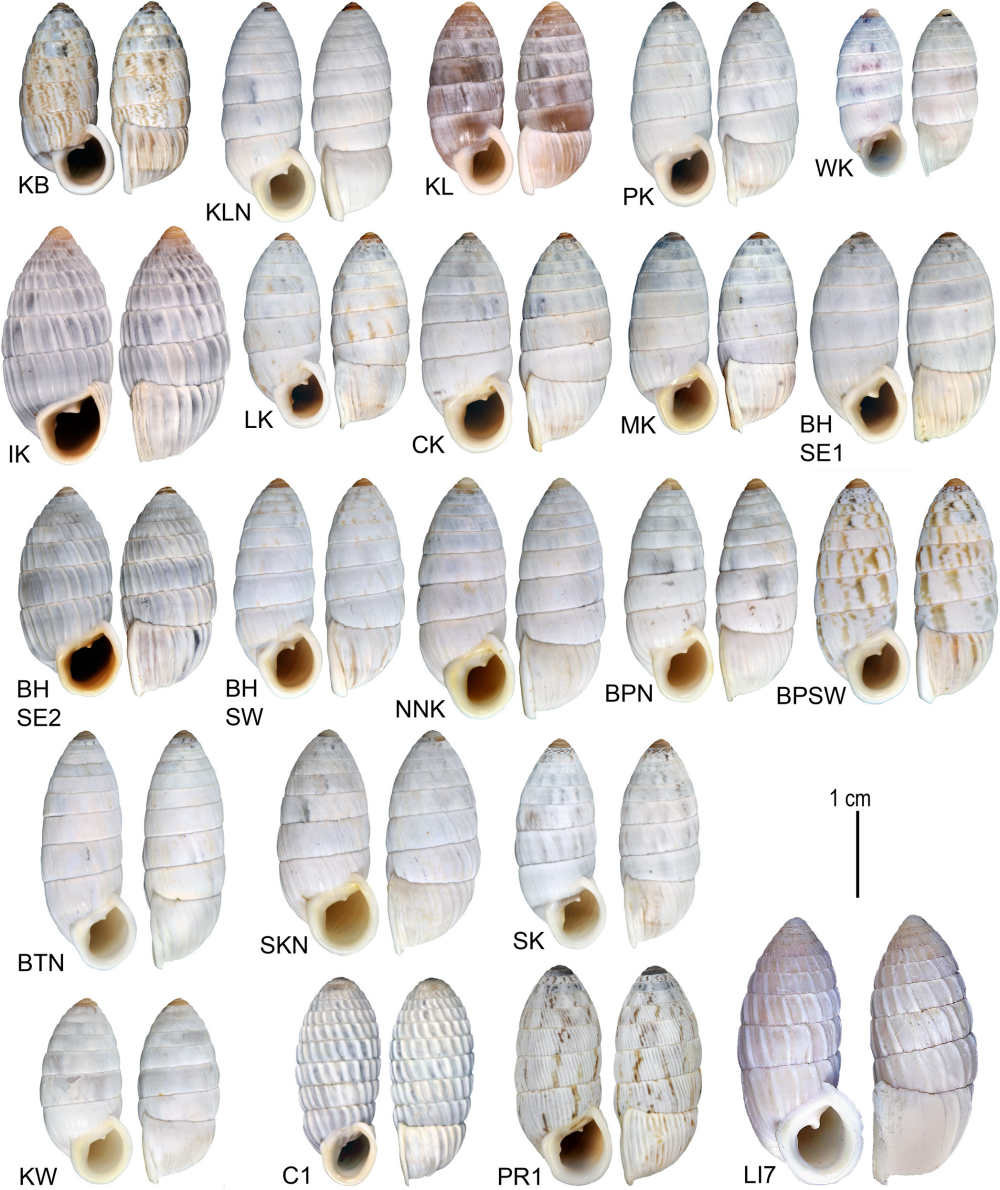
Representative specimens of *Cerion* populations used in this study. Labels correspond to station letter designations in Table 1.

Neither *Cerion incanum* nor *Cerion casablancae* are endangered or protected species. Populations of *Cerion* were sampled from 18 locations between Key Biscayne and Key West (Table 1, Figs 1 and 2). Sampling sites were selected to span the known geographic range of the species based on historical museum records, and to include the type localities for each of the four named subspecies of *Cerion incanum* [*C*. *incanum incanum* (Leidy, 1851)–Key West; *C*. *incanum fasciatum* (Binney, 1859) [21]–Key Biscayne; *C*. *incanum saccharimeta* (Pilsbry, & Vanatta, 1899) [22]–Sugarloaf Key; *C*. *incanum vaccinum* (Pilsbry, 1902) [20]–Vacca Key] that had been proposed on the basis of shell morphology. Our sample from Indian Key is comprised of the descendants of 500 specimens of *Cerion cassablancae* Bartsch, 1920 [14], that were introduced to the Key on June 1, 1912, after a survey determined that no native *Cerion* were present on the island [28]. The following day, Bartsch introduced an additional 500 individuals of *Cerion cassablancae* onto the eastern portion of Bahia Honda Key, where they survived for multiple generations [14, 28]. During a regular survey of this colony on September 23, 1931, Bartsch [29] was surprised to discover *Cerion incanum* on Bahia Honda Key, as well as the presence of hybrids between *Cerion incanum* and *C*. *cassablancae* near the site of his introduction. Our sample from the southwestern portion of Bahia Honda Key is comprised of *Cerion incanum*, while specimens from the southeastern shore of Bahia Honda are a sample of the hybrid population [*Cerion incanum* X *C*. *cassablancae*].

**Table 1:** Florida Keys populations of *Cerion* sampled for this study. The numbers of individuals sequenced from each population for the mitochondrial cytochrome *c* oxidase 1 and 16S rDNA genes, the numbers of different haplotypes within each population for each of the partial gene sequences, as well as for the concatenated sequences are provided. Samples from the type localities of named taxa are in bold. GenBank accession numbers for the various haplotypes of *Cerion incanum*, *C*. *cassablancae* and their hybrids are provided in Appendix 1.

					COI	16S	Concatenated
Taxon	Station	Locality	USNM #	n =	# haplotypes	# haplotypes	# haplotypes
***Cerion incanum fasciata***	KB	Key Biscayne	1201700	21	3	4	5
*Cerion incanum*	KLN	Key Largo, North	1212784	28	2	3	3
*Cerion incanum*	KL	Key Largo	1201711	23	3	2	3
*Cerion incanum*	PK	Plantation Key	1212803	31	7	11	12
*Cerion incanum*	WK	Windley Key	1212807	28	8	4	14
*Cerion casablancae*	IK	Indian Key	1175018	6	4	3	4
*Cerion incanum*	LK	Long Key	1212814	29	3	3	3
***Cerion incanum vaccinum***	CK	Crawl Key	1212819	30	5	6	11
*Cerion incanum*	MK	Missouri Key	1201707	22	2	1	2
*Cerion incanum x casablancae*	BHSE	Bahia Honda Key, Southeast	1212826	30	14	12	19
*Cerion incanum*	BHSW	Bahia Honda Key, Southwest	1212831	29	1	2	2
*Cerion incanum*	NNK	No Name Key	1212836	32	7	7	8
*Cerion incanum*	BPN	Big Pine Key, North	1212840	30	14	10	18
*Cerion incanum*	BPSW	Big Pine Key, Southwest	1212844	31	10	7	12
*Cerion incanum*	BTN	Big Torch Key, North	1212848	31	11	7	14
***Cerion incanum saccharimeta***	SKN	Sugarloaf Key, North	1212856	31	14	14	20
*Cerion incanum saccharimeta*	SK	Sugarloaf Key	1201710	27	12	5	12
***Cerion incanum incanum***	KW	Key West	1201706	27	6	3	7
			TOTAL	486	126	104	169
OUTGROUPS							
*Cerion uva*	C1A	Schaarlo, Curaçao		1	KJ624975	KJ636144	
*Cerion striatellum*	PR1B	Tamarindo Beach, Puerto Rico		1	KJ934716	KJ636083	
*Cerion caerulescens*	L17B	Long Key, Bahamas		1	KJ934722	KJ636089	

Samples were collected along the sides of public roads or along public beaches, not on private property. No specific permissions were required for these localities. Specimens were collected at each location, generally on and under low vegetation and rocks in close proximity to water, and transported living to the laboratory at the National Museum of Natural History, Smithsonian Institution. All specimens collected for this study are part of the collections of the National Museum of Natural History, Smithsonian Institution. Catalog numbers are provided in Table 1 [http://collections.nmnh.si.edu/search/iz (search by catalogue number in Table 1)].

### DNA extraction, PCR amplification and sequencing

DNA was obtained from a subset of the individuals (n = 6–31) from each population by removing the dorsum of the shell using a Wizard Model 100 Saw with a fine diamond blade and dissecting half the buccal mass (~20 mg) from the animal. Genomic DNA was extracted using an Autogen GENE PREP965 (Autogen) employing the manufacturer's mouse-tail tissue protocol with a final elution volume of 100 μL. The shells were retained as voucher specimens and individually identified with a number suffix to the USNM catalog number assigned to each population. [Images of the sequenced specimens are appended to sample catalog records: http://collections.nmnh.si.edu/search/iz/ (search by catalogue number in Table 1) and also appear on the Cerion website: http://invertebrates.si.edu/cerion/ under the taxon names and station numbers shown in Table 1].

Portions of two mitochondrial genes were amplified: a 655 bp region of the cytochrome c oxidase I gene using the primers HCO1490 AND LCO2198 [30] and a 510 bp region of the 16S ribosomal gene using the primers 16S-ar and 16S-br [31]. PCR amplifications were performed in 20 μL volumes that contained: 12.9 μL ddH_2_O, 2 μL 10x NH_4_ reaction buffer (Bioline) 1.2μL of 50mM MgCl_2_ (Bioline), 1 μL dNTPs, 0.5 μL of 100x BSA (BioLab), 0.6 μL of 10μM forward primer (Integrated DNA Technologies, Inc.), 0.6 μL of 10μM reverse primer (Integrated DNA Technologies, Inc.), 0.2 μL of Biolase DNA polymerase (Bioline) and 1 μL of extracted genomic DNA. For COI, the amplification protocol for the thermocycler (Applied Biosystems, Life Technologies) was as follows: initial denaturation at 95°C for 3 minutes, followed by 45 cycles of denaturation at 94°C for 30 seconds, annealing at 45°C for 45 seconds and extension at 72°C for 2 minutes, with a final extension at 72°C for 5 minutes. For the 16S rDNA gene: initial denaturation at 95°C for 3 minutes, followed by 40 cycles of denaturation at 95°C for 45 seconds, annealing at 48°C for 45 seconds, and extension at 72°C for 2 minutes, with a final extension at 72°C for 5 minutes. Resulting PCR products were visualized by agarose gel electrophoresis (1.5% agarose) and purified with ExoSAP-IT (Affymetrix) according to manufacturer’s protocols, prior to sequencing.

Sequencing reactions were performed in 10 μL volumes that contained: 6.25 μL ddH_2_O, 1.75 μL 5x sequencing buffer, 0.5 μL primer, 0.5 μL Big Dye, and 1 μL of purified PCR product, and run in the thermal cycler for 30 cycles of 95°C for 30 seconds, 50°C for 30 seconds, and 60°C for 4 minutes, then held at 12°C. Reaction products were purified using Millipore Sephadex plates (Millipore, Billerica, MA) using manufacturer's protocols and sequenced on an ABI 3730XL automated DNA Analyzer (Applied Biosystems and Hitachi, Ltd.). Geneious 7.1.2 (Biomatters, Ltd.) was used to visualize, trim, edit and assemble contigs from forward and reverse sequences. Individual sequences are identified by the Station letter designation (Table 1) followed by the specimen number (e.g., KB3 refers to the sequence derived from specimen 3 from the Key Biscayne population and refers to specimen 3 of USNM 1201700). The program TCS v. 1.21 [32] was used to sort identical sequences for each gene into haplotypes, which are listed in Appendix 1 together with their corresponding specimens and GenBank (NCBI) accession numbers.

### Species delimitation

The Automatic Barcode Gap Discovery (ABGD) method aggregates sequences into candidate species based on gaps in the distribution of pairwise differences that correspond to gaps between intraspecific and interspecific divergences [33]. It is one of several methods for species discovery and delimitation using molecular techniques recently evaluated [34] and found to be among the more conservative and objective.

Samples are parsed into a decreasing number of groups in a series of recursive partitions. The ABGD method (http://wwwabi.snv.jussieu.fr/public/abgd/; default parameters) was applied to a matrix of pairwise distances (Jukes-Cantor model, pairwise deletion of missing data) produced for the COI and 16S datasets using MEGA v. 5.05 [35] in order to evaluate hypotheses of species delimitation.

### Phylogenetic analyses

Alignments of COI and 16S were obtained using MAFFT [36] for 16S and MUSCLE (Multiple Sequence Comparison by Log-Expectation) [37] for COI. The aligned sequences were concatenated using Geneious version 7.1.2 [38]. In order to expedite the analyses, the program TCS v. 1.21 [32] was used to identify and collapse individual concatenated sequences at each locality. In these analyses, collapsed sequences are identified by an exemplar sequence followed by an "x" and the number of individuals with that identical sequence (e.g., NNK1 x 15 indicates that there are 15 specimens with a concatenated sequence identical to sample NNK1).

A best-fit model of nucleotide sequence evolution (compatible with MrBayes) and partitioning arrangement for each locus was determined by PartitionFinder v.1.1.1 [39, 40] using the “greedy” search and AICc scheme options. The GTR+G model was chosen for 16S, and models for each codon position (1^st^: GTR+I, 2^nd^: F81, and 3^rd^: GTR+G) where chosen for COI.

Phylogenetic analyses were performed on a concatenated dataset (16S+COI) using Bayesian Inference (BI) performed with MrBayes 3.1.2 [41] and Maximum Likelihood (ML) with RAxML [42]. All analyses were run on the Topaz computing cluster, which is maintained by the Laboratories of Analytical Biology at the National Museum of Natural History. BI analysis was carried out for 10 million generations with two independent runs, each with four chains, and with trees sampled every 1000th generation. Model parameters (tratio, statefreq, shape, pinvar) were unlinked among partitions, and the rate prior (prset ratepr) was set to “variable”. Convergence was determined when the average standard deviation of split frequencies was <0.01 and the potential scale reduction factor (PSRF) was 1.00. In addition, TRACER v1.5 [43] was used to verify that an adequate amount of trees were sampled from the posterior distribution, and to confirm the stationarity of the runs. To calculate posterior probabilities, a “burn-in” of 25% of the total trees sampled per run adequately removed trees prior to convergence. ML options for RAxML included the GTRGAMMA model of nucleotide evolution (-m), rapid bootstrap analysis and search for best-scoring ML tree (-f m), and 1000 bootstrap replicates.

### Haplotype networks

The program TCS v. 1.21 [32] was used to estimate haplotype networks for the COI and for the 16S genes (with outgroups removed) under default settings (gaps treated as a fifth base, 95% confidence interval for statistical parsimony). Correlations between haplotypes and the specimens at the various localities are provided in Appendix 1.

## Results

Specimens from each of the sampled populations (Table 1, Figs 1 and 2) were sorted to the named subspecies of *Cerion incanum* (Table 2) based on a comparison of their shells to typical specimens of the respective subspecies (Fig 1A–1D). In many cases, these attributions were provisional, as the phenotypes exemplified by the type specimens are extreme. Morphological features on which the subspecies are based are less pronounced in the majority of specimens. Many individuals have phenotypes that are intermediate between subspecies. Some populations have significant proportions of three (BHSW) or all four phenotypes (BPSW).

**Table 2:** Distribution of shell phenotypes within and among the sampled *Cerion* populations as % of each population. Images of specimens are appended to their catalog records http://collections.nmnh.si.edu/search/iz/ (search by catalogue number in Table 1).

Population	n =	*Cerion incanum incanum*	*Cerion incanum saccharimeta*	*Cerion incanum vaccinum*	*Cerion incanum fasciata*	*Cerion casablancae*
KB	32	75	0	0	25	0
KLN	32	97	0	3	0	0
KL	24	96	0	4	0	0
PK	32	100	0	0	0	0
WK	32	94	0	6	0	0
IK	12	0	0	0	0	100
LK	32	97	0	3	0	0
CK	32	94	0	0	0	0
MK	32	100	0	0	0	0
BHSE	32	35	6	3	0	56
BHSW	32	72	6	0	22	0
NNK	32	91	9	0	0	0
BPN	32	81	19	0	0	0
BPSW	32	43	16	16	25	0
BTN	32	75	9	0	16	0
SKN	28	50	47	0	3	0
SK	32	84	16	0	0	0
KW	30	100	0	0	0	0

Sequences for portions of the COI and 16S rDNA genes were obtained for 450 individuals of *Cerion incanum* spanning the entire geographic range of this species, 30 individuals from a *C*. *incanum* x *C*. *casablancae* hybrid population along the southeastern coast of Bahia Honda Key, and 6 individuals of *Cerion casablancae* from the introduced colony on Indian Key (Table 1).

### Data analysis

For the COI gene, an alignment of 655 bp, corresponding to positions 39–693 of the complete COI gene in *Cerion incanum*, was produced after primers were trimmed. The COI alignment contained 134 (20.4%) variable and 116 (17.7%) parsimony informative sites. When converted to amino acids, only 23 (10.6%) of 218 positions were variable, and 17 (7.8%) parsimony informative. The 16S gene alignment contained 510 bp corresponding to positions 564–1068 of this gene in *C*. *incanum*. Of these, 84 (16.5%) were variable and 70 (13.7%) were parsimony informative. The concatenated data set consisted of 1165 positions, of which 326 (30.0%) were variable and 233 (20.0%) were parsimony informative.

**Fig 3:**
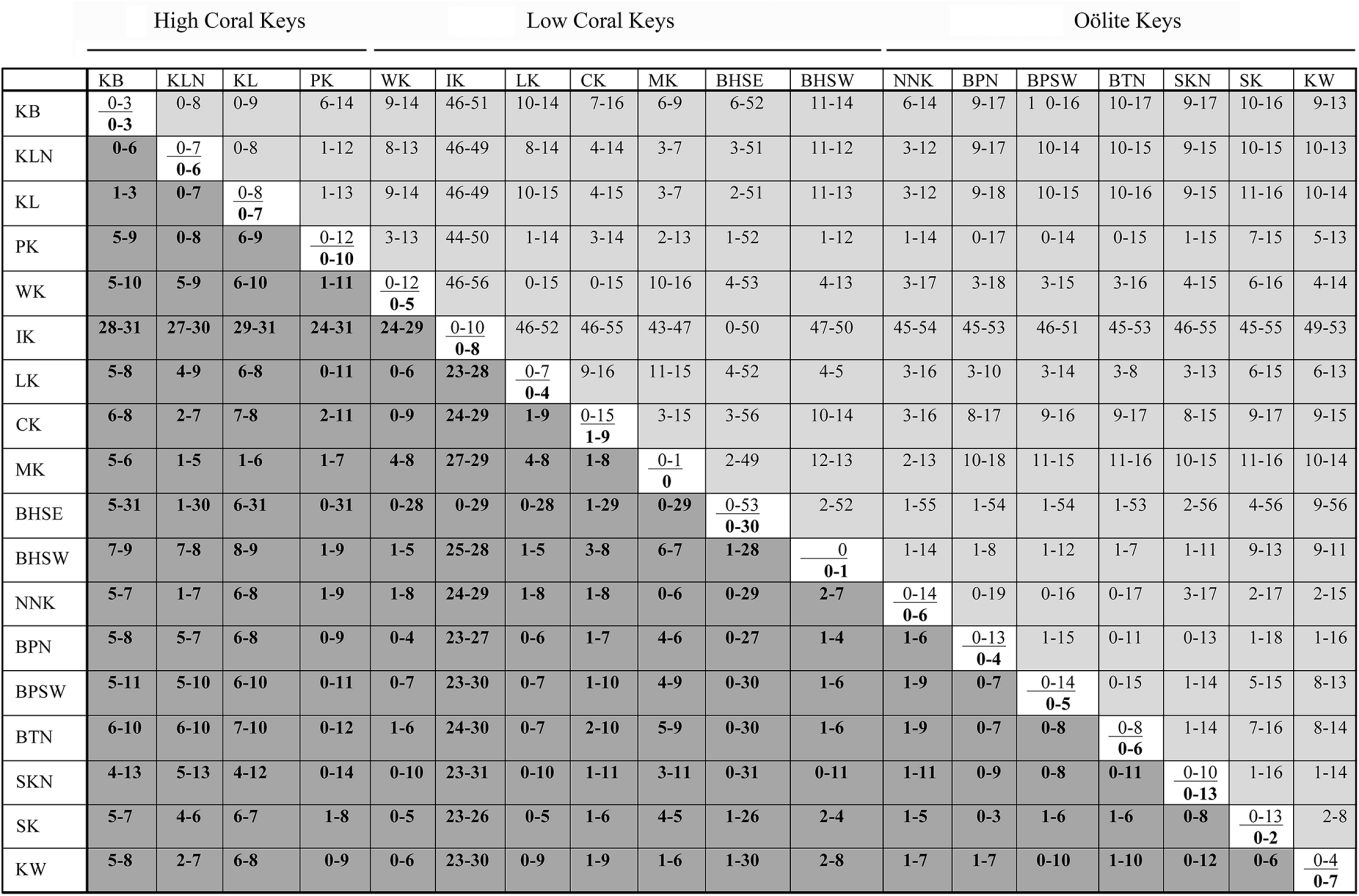
Nucleotide differences within and between *Cerion* populations in the Florida Keys. Within population differences are shown on the diagonal, with the total number of nucleotide differences in the cytochrome C oxidase 1 gene (COI) (of 655 bp) above the line and the **total number of nucleotide differences in the 16S rDNA gene** (of 510 bp) below the line. Differences in the COI gene between populations are shown above the diagonal, **differences in the 16S rDNA gene** between populations are shown below the diagonal.

Only the population from the southwestern portion of Bahia Honda Key (BHSW) was monomorphic for the COI gene, and only the Missouri Key population (MK) was monomorphic for the 16S gene (Table 1, Fig 3). For all *Cerion incanum* populations spanning the Florida Keys, the maximum sequence diversity for COI was 2.14% within populations, and 2.90% between populations. For the 16S gene, the values were 2.55% within populations and 2.75% between populations. Maximum sequence diversity within the introduced population of *Cerion casablancae* on Indian Key (IK) was 1.53% for the COI gene and 1.56% for the 16S gene. Differences between *Cerion incanum* and the Indian Key population of *C*. *casablancae* ranged between 6.56% and 8.55% for COI gene and between 4.51% and 6.08% for the 16S gene. Levels of sequence diversity within the *C*. *incanum* x *C*. *casablancae* hybrid population in the southeastern region of Bahia Honda Key (BHSE) reached 8.09% for COI and 5.88% for 16S, but were clearly bimodal [ranges for COI = 0.00–3.97% and 7.33–8.09% / for 16S = 0.00–2.16% and 4.90–5.88%], corresponding to comparisons between homologous and heterologous mitogenomes respectively.

Within *Cerion incanum*, greatest haplotype diversity for both COI and 16S genes occurred in the Oölite Keys (NNK to KW) [ = 10.6 for COI, 7.6 for 16S] far greater than in the High Coral Keys [ = 3.8 for COI, 5.0 for 16S] or the Low Coral Keys [ = 3.8 for COI, 3.2 for 16S].

### Species delimitation

Results of Automatic Barcode Gap Discovery (ABGD) analyses of the COI and 16S datasets are shown in Table 3. In both the COI and 16S analyses, the first two partitions segregated nearly all haplotypes as separate groups. All *Cerion incanum* haplotypes were combined into a single group in the third and all subsequent partitions in both analyses. Indian Key samples of *C*. *casablancae* were progressively grouped with some to all *C*. *incanum* x *C*. *casablancae* hybrids with mitogenomes derived from *C*. *casablancae* in partitions 3–6, and with *C*. *incanum* in partitions 7–10, in analyses of both genes. The outgroup taxa remained distinct in all partitions in all analyses.

**Table 3:** Results of automatic barcode gap discovery analyses of distance matrices for the COI and 16S datasets. *Cerion incanum* haplotypes are numbered, *Cerion casablancae* haplotypes (from Indian Key) are prefixed with the letter C.

Partition	No. of Groups found COI gene (117 haplotypes)	No. of Groups found 16S gene (81 haplotypes)	Prior maximum distance
1	116	75	P = 0.001000
2	116	75	P = 0.001668
3	8	8	P = 0.002783
4	8	5	P = 0.004642
5	6	5	P = 0.007743
6	5	5	P = 0.012915
7	4	4	P = 0.021544
8	4	4	P = 0.035938
9	4	4	P = 0.059948
10	4	4	P = 0.100000

Composition of Groups resulting from analysis of the COI sequences (117 haplotypes). 116 Groups = *C*. *uva* + *C*. *striatellum* + *C*. *caerulescens* + 1 + 2 + 3 + 4 + 5 + 6 + 7 + 8 + 9 + 10 + 11 + 12 + 13 +14 + 15 + 16 + 17 + 18 + 19 + 20 + 21 + 22 + 23 + 24 + 25 + 26 + 27 + 28 + 29 + 30 + 31 + 32 + 33 + 34 + 35 + 36 + 37 + 38 + 39 + 40 + 41 + 42 + 43 + 44 + 45 + 46 + 47 + 48 + 49 + 50 + 51 + 52 + 53 +54 + 55 + 56 + 57 + 58 + 59 + 60 + 61 + 62 + 63 + 64 + 65 + 66 + 67 + 68 + 69 + 70 + 71 + 72 + 73 + 74 + 75 + 76 + 77 + 78 + 79 + 80 + (81+ 85) + 82 + 83 + 84 + 86 + 87 + 88 + 89 + 90 + 91 + 92 + 93 + 94 + 95 + 96 + 97 + 98 + 99 +100 + 101 + 102 + 103 + 104 + 105 + 106 + 107 + 108 + 109 + 110 C1 + C2 + C3 + C4

8 Groups = *C*. *uva* + *C*. *striatellum* + *C*. *caerulescens* + (1–105) + 106 + (107 + 108) + (109 + 110 + C1) + (C2–C4)

6 Groups = *C*. *uva* + *C*. *striatellum* + *C*. *caerulescens +* (1–105) + 106 + (107–110 + C1–C4)

5 Groups = *C*. *uva* + *C*. *striatellum* + *C*. *caerulescens +* (1–105) + (106–110 + C1–C4)

4 Groups = *C*. *uva* + *C*. *striatellum* + *C*. *caerulescens +* (1–110 + C1–C4)

Composition of Groups resulting from analysis of the 16S sequences (81 haplotypes). 75 Groups = *C*. *uva* + *C*. *striatellum* + *C*. *caerulescens* + (1 + 7) + (2 + 5 + 6) + 3 + 4 + 8 + 9 + 10 + 11 + 12 + 13 + 14 + 15 + 16 + 17 + 18 + 19 + (20 + 64) + 21 + 22 + 23 + 24 + 25 + (26 + 63) + 27 + 28 + 29 + 30 + 31 + 32 + 33 + 34 + 35 + 36 + 37 + 38 + 39 + 40 + 41 + 42 + 43 + 44 + 45 + 46 + 47 + 48 + 49 + 50 + 51 + 52 + 53 + 54 + 55 + 56 + 57 + (58 + 62) + 59 + 60 + 61 + 65 + 66 + 67 + 68 + 69 + 70 + 71 + 72 + 73 + 74 + 75 + C1 + C2 + C3

8 Groups = *C*. *uva* + *C*. *striatellum* + *C*. *caerulescens* + (1–71) + (72+73+74 + C3) + 75 + C1 + C2

5 Groups = *C*. *uva* + *C*. *striatellum* + *C*. *caerulescens* + (1–71) + (72+73+74 + 75 + C1+ C2 + C3)

4 Groups = *C*. *uva* + *C*. *striatellum* + *C*. *caerulescens* + (1–75 + C1–C3)

### Phylogenetic analyses

**Fig 4:**
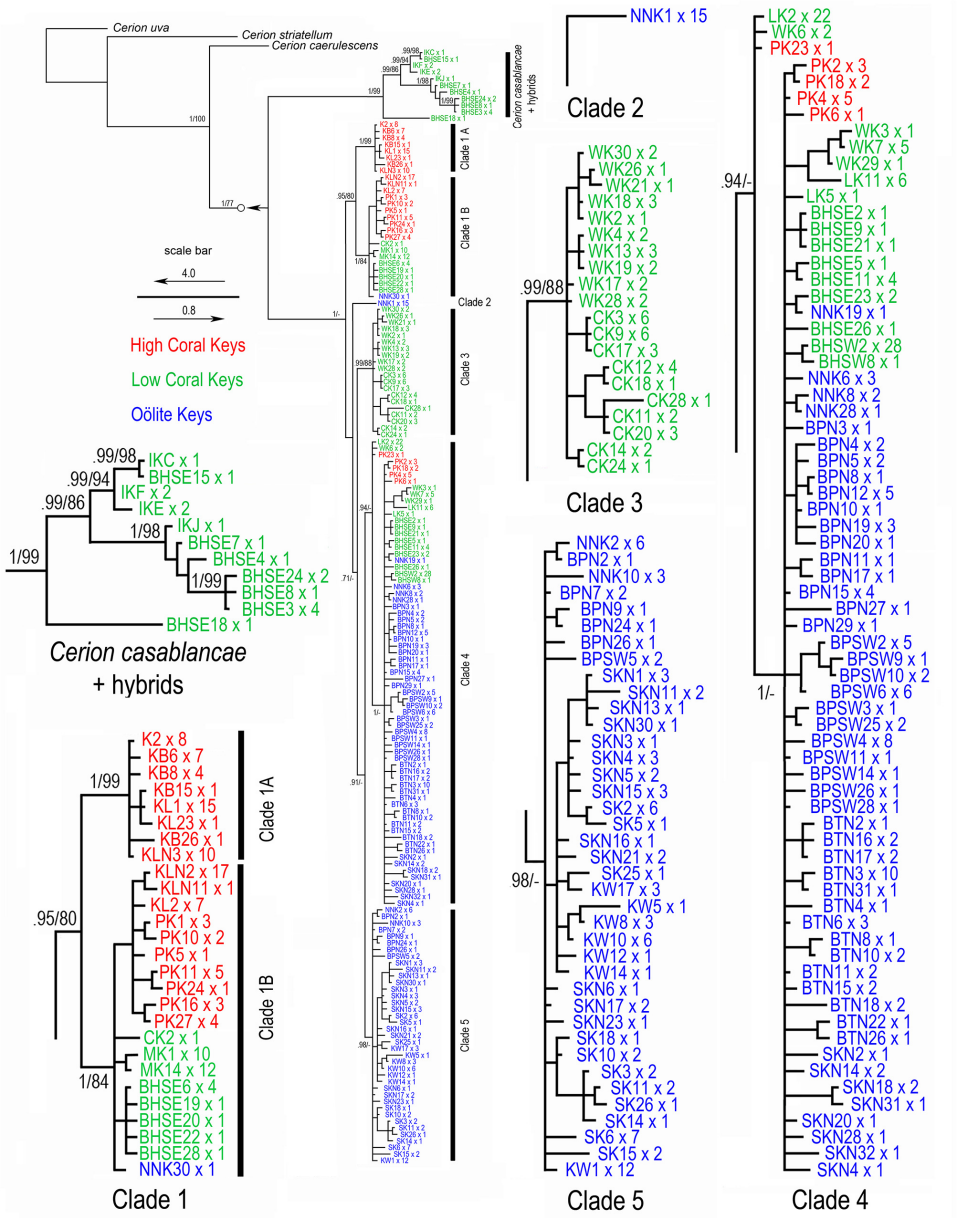
Phylogenetic relationships among the *Cerion* of the Florida Keys. Result of Bayesian analysis of the concatenated data including 16S and COI sequences. Numbers on nodes indicate BI posterior probabilities and ML bootstrap values respectively. Geographic regions are color coded.

Both Bayesian and Maximum Likelihood analyses of the concatenated (16S + COI) dataset resulted in a tree that resolved the relationships among the outgroup taxa and the *Cerion* of the Florida Keys with high support and partitioned all *Cerion* samples from the Florida Keys into two clades: the *casablancae* clade, which included all specimens of *C*. *casablancae* from Indian Key, and a subset of *C*. *incanum* x *C*. *casablancae* hybrids from the Bahia Honda SE population with mitochondrial genomes derived from *C*. *casablancae*; and the *incanum* clade, which included all specimens of *Cerion incanum*, including all *C*. *incanum* x *C*. *casablancae* hybrids from the Bahia Honda SE population with mitochondrial genomes inherited from *C*. *incanum*. [Mitochondrial genomes are maternally inherited]. The phylogenetic tree obtained using Bayesian Likelihood analysis of the concatenated (16S + COI) dataset is shown in Fig 4. Posterior probabilities (PP) as well as bootstrap values (BV) derived from Maximum Likelihood analysis are indicated on the main nodes [PP ≥ 0.90, BV ≥ 70%].

**Fig 5:**
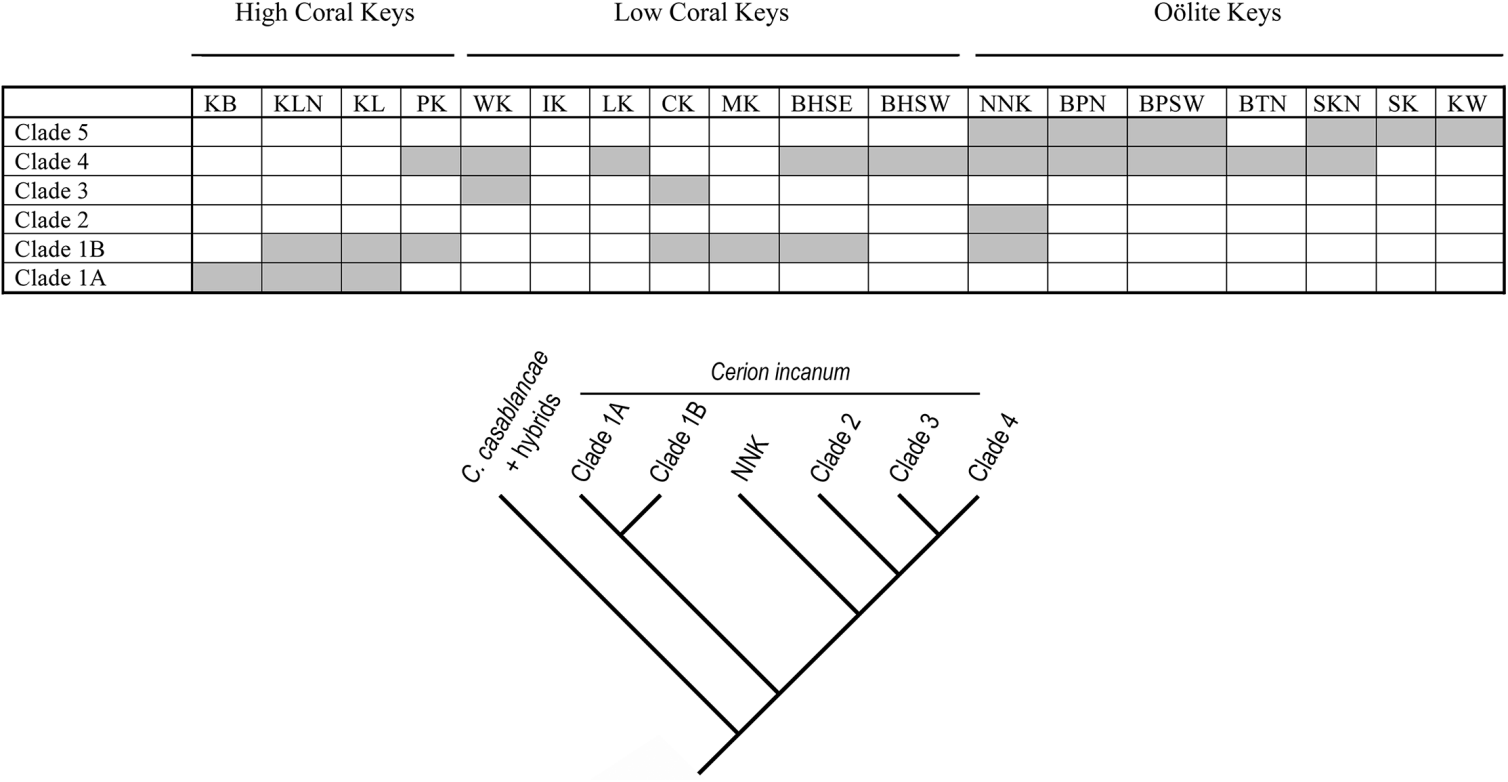
Geographic distribution of haplotypes present in the major clades of *Cerion incanum* in the Florida Keys identified in Fig 4.

Five major clades were resolved within the *incanum* clade (Fig 5). Clade 1 is the most basal, and was composed of two subclades. Clade 1A, consists of 8 haplotypes and 47 individuals, all from the High Coral Keys. It includes all the specimens from Key Biscayne and representatives of both populations from Key Largo, but none from Plantation Key. Clade 1B is larger (19 haplotypes, 76 individuals) and includes specimens from the High Coral Keys (Key Largo to Plantation Key), the southwestern Low Coral Keys, as well as a single individual from No Name Key from the Oölite Keys. The next most basal group consists of a single haplotype occurring in 15 individuals from No Name Key. Clade 3 is composed of 20 haplotypes and 48 individuals, all from Windley Key and Crawl Key. Both are Low Coral Keys. No individuals from Long Key, situated between these two Keys, are included in this clade. Clade 4 is the largest (71 haplotypes, 191 individuals) and most geographically widespread clade, extending from Plantation Key the southernmost of the High Coral Keys, and spanning the Low Coral Keys as well as most of the Oölite Keys as far as Sugar Loaf Key. Clade 5 (39 haplotypes, 92 individuals) is the most derived clade. It is confined to the Oölite Keys.

### Haplotype networks

The program TCS sorted 486 COI sequences from *Cerion incanum*, *C*. *casablancae* and their hybrids into 114 haplotypes. Of these, 48 (42%) haplotypes were represented by single individuals (singletons) and 8 (7%) were present in more than one population. The corresponding 16S sequences included 78 haplotypes, of which 30 (38%) were singletons and 15 (19%) present in multiple populations (Appendix 1).

**Fig 6:**
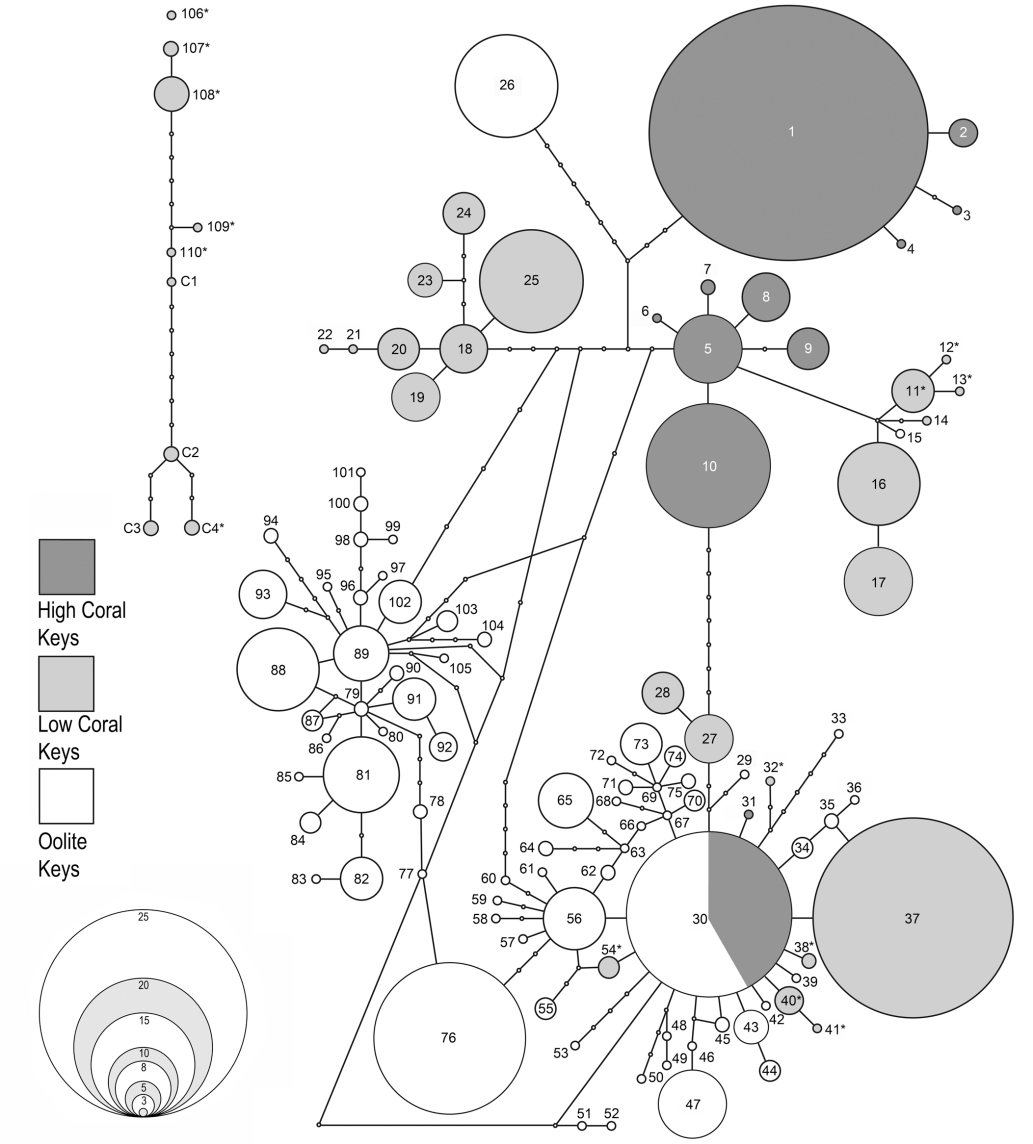
TCS network based on COI haplotypes of *Cerion* of the Florida Keys. Haplotypes are sequentially numbered. In instances where identical haplotypes occur in more than one region, circles are proportionally shaded. * denotes haplotypes present in the BHSE population that includes *C*. *incanum* x *C*. *casablancae* hybrids. (See Appendix 1 for correlation of network haplotype numbers with individuals and populations as they appear in the Bayesian analysis.)

**Fig 7:**
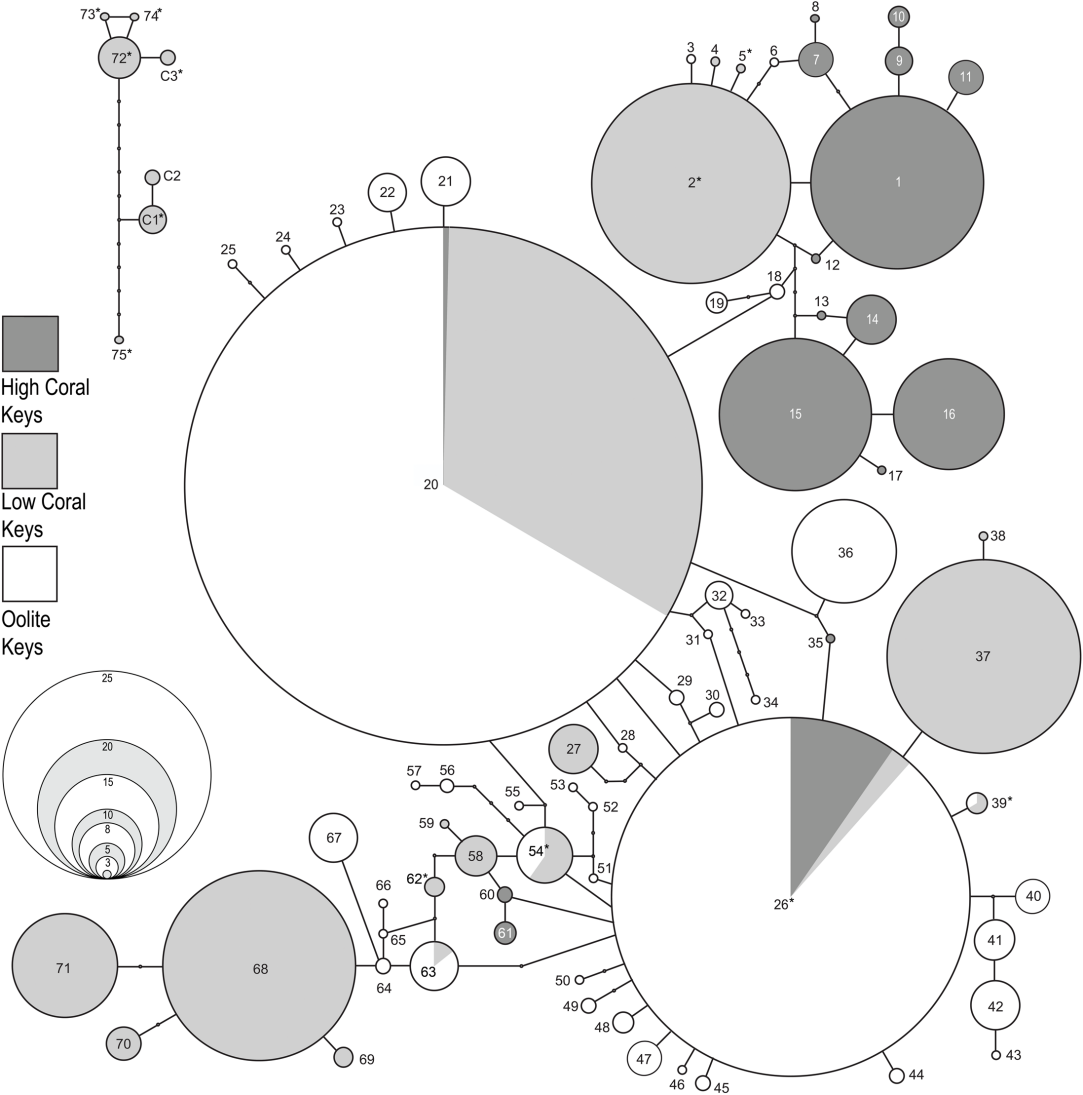
TCS network based on 16S haplotypes of *Cerion* of the Florida Keys. Haplotypes are sequentially numbered. In instances where identical haplotypes occur in more than one region, circles are proportionally shaded. * denotes haplotypes present in the BHSE population that includes *C*. *incanum* x *C*. *casablancae* hybrids. (See Appendix 1 for correlation of network haplotype numbers with individuals and populations as they appear in the Bayesian analysis.)

The haplotype network for the COI gene (95% confidence interval, connection limit = 11 steps) is shown in Fig 6, the network for the 16S gene (95% confidence interval, connection limit = 9 steps) in Fig 7. Haplotypes from the High Coral Keys, Low Coral Keys and Oölite Keys are shaded in different tones. Haplotypes present in more than one of these areas are shaded proportionally. In both analyses, haplotypes occurring in *Cerion casablancae* and *C*. *incanum* x *C*. *casablancae* hybrids with mitogenomes derived from *C*. *casablancae* form a network that is separate from the network of haplotypes occurring in *C*. *incanum* and *C*. *incanum* x *C*. *casablancae* hybrids with mitogenomes derived from *C*. *incanum*, indicating that it would require more than 11 steps to join the COI networks, and more than 9 steps to join the 16S networks. In one case, the COI haplotype (Haplotype 106) was more than 11 steps from either network.

**Fig 8:**
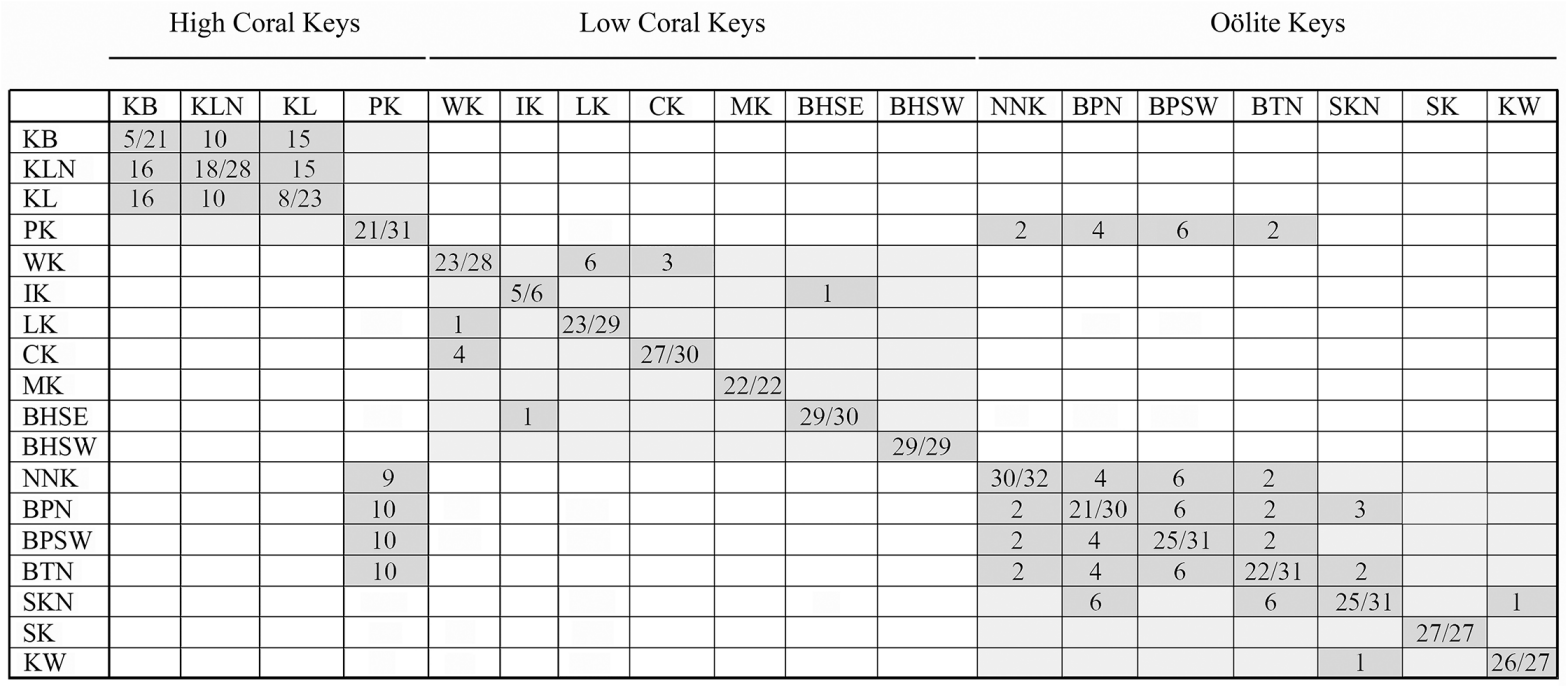
COI haplotypes shared among Keys. Diagonal shows the number of specimens with haplotypes endemic to the Key / number of individuals sequenced from the Key for COI. Rows indicate number of specimens on other Keys that have haplotypes that occur on specified Key. Columns indicate number of specimens on specified Key that share a haplotype with other Keys.

With the exception of the Plantation Key population, COI haplotypes tended to be geographically restricted. All COI haplotypes occurring in samples from the northern High Coral Keys (KB, KLN, KL) were shared by and confined to these three populations, and were not represented elsewhere in the Keys (Fig 8). Similarly, COI haplotypes occurring in the Oölite Keys were restricted to these Keys. A notable and anomalous exception was Plantation Key, which shared a single wide-ranging COI haplotype only with four of the Oölite Keys. Haplotypes of COI occurring in the Low Coral Keys were limited to this group of islands. Excluding the special case of the *C*. *casablancae* haplotype shared by Indian Key and the Bahia Honda SE population, which was due to well-documented introductions, only Windley Key, Long Key and Crawl Key had COI haplotypes in common. Except for Key Biscayne (KB) and the population from southern Key Largo (KL) the majority (≥64%) of COI haplotypes occurring in each of the sampled populations were endemic to that population. Other than the wide-ranging Haplotype 30, and Haplotype 31 (which differs by a single step), both from Plantation Key, all COI haplotypes occurring on the High Coral Keys are directly interconnected on the COI Haplotype network by 1–10 steps. This High Coral Key Network is closest (2 steps) to a cluster of haplotypes present only on the southwestern Low Coral Keys (CK, MK, BHSE) and No Name Key. Eight steps separate it from two COI haplotype clusters that are present in the northeastern Low Coral Keys. COI haplotypes occurring in the Oölite Keys are also all interconnected by 1–25 steps in a single, complex network with reticulate patterns of multiple loops that may include the wide-ranging Haplotype 30, which occurs on the Oölite Keys as well as on the High Coral Keys. In contrast, COI haplotypes present on the Low Coral Keys occur in several clusters that are not interconnected. Some appear derived from haplotypes present on the High Coral Keys, others from haplotypes of the Oölite Keys. A minimum of 8 steps separates the High Coral Key Network from the network of COI haplotypes of the Oölite Keys.

**Fig 9:**
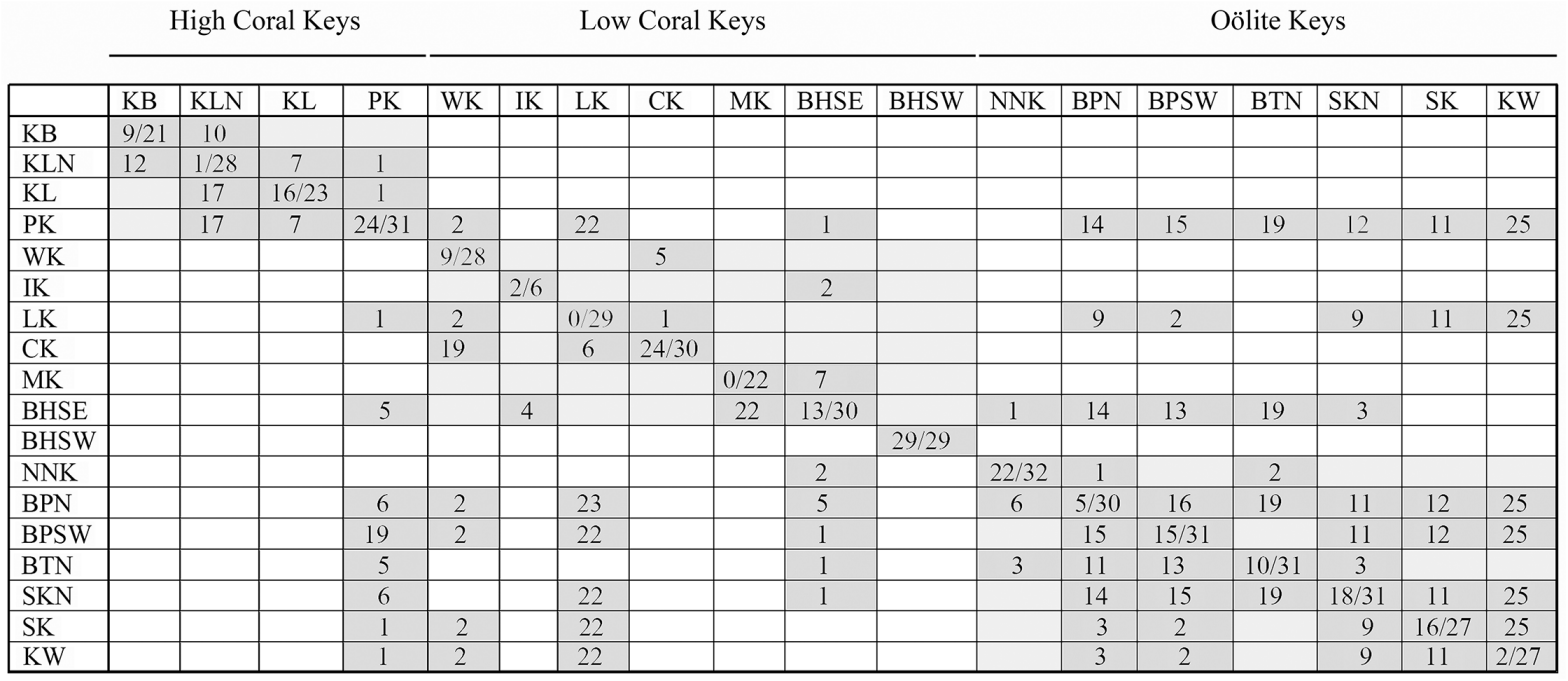
16S haplotypes shared among Keys. Diagonal shows the number of specimens with haplotypes endemic to the Key / number of individuals sequenced from the Key for 16S. Rows indicate number of specimens on other Keys that have haplotypes that occur on specified Key. Columns indicate number of specimens on specified Key that share a haplotype with other Keys.

As with the COI haplotypes, most of the 16S haplotypes occurring on the High Coral Keys are endemic to the High Coral Keys (Fig 9), and differ by 1–10 mutational steps (Fig 7). The exceptions are Haplotype 26, which occurs on Plantation Key in addition to several Oölite Keys, as well as Haplotypes 35, 60 and 61 which are within 2 mutational steps of Haplotype 26 and endemic to Plantation Key. Most of the 16S haplotypes occurring on the Oölite Keys are interconnected, often through complex linkages that may include one or more haplotypes (eg., Haplotypes 20, 26, 54, 63) with ranges that extend beyond the Oölite Keys. 16S haplotypes represented on the Low Coral Keys are more widely distributed, and few are interconnected. Unlike the COI haplotypes, a much smaller number of populations sampled had a majority of 16S haplotypes that were endemic to a Key.

### The hybrid population on Bahia Honda Key

Thirty individuals from the *C*. *incanum* x *C*. *casablancae* hybrid population inhabiting the southeastern coast of Bahia Honda Key were sequenced. Of these, 19 had mitogenomes derived from *Cerion incanum* and 11 from *C*. *casablancae*. The COI haplotypes derived from *C*. *incanum* mitogenomes were all endemic to the BHSE population and were within 3 mutational steps of haplotypes present on MK, NNK or from a wide ranging haplotype present on the Oölite Keys and Plantations Key.

Of the specimens with the *C*. *casablancae* derived mitogenomes, one shared a COI haplotype present in a specimen of *C*. *casablancae* from Indian Key, the rest had COI haplotypes that were endemic to the BHSE population. Most differed by 1–8 bases from Indian Key specimens of *C*. *casablancae*, but one could not be connected to either of the mitogenome networks within the 95% confidence interval (11 steps).

The 16S haplotypes present in specimens with *C*. *casablancae* mitogenomes were either *C*. *casablancae* or endemic to the BHSE population and within 6 mutational steps from *C*. *casablancae* haplotypes. Specimens with mitogenomes inherited through *C*. *incanum* maternal lineages had more diverse 16S haplotypes. Four were endemic to the BHSE population, 7 also occurred on neighboring MK, 2 on NNK, 5 on BPN and one was shared with populations ranging from PK to SKN.

## Discussion

*Cerion incanum* is endemic to the Florida Keys, ranging from Key Biscayne to Key West, and the only species of *Cerion* native to the archipelago. Like most *Cerion*, it forms dense local colonies that are not widespread, and does not occur on every Key. A major factor affecting dispersal among islands is the transport by hurricanes of vegetation to which these animals are attached [1, 22, 44]. Prior to his initial series of experiments, Bartsch [28, 29] surveyed many of the Keys and introduced Bahamian species of *Cerion* only onto islands on which *C*. *incanum* was not found. Indian Key and Bahia Honda Key were among the uninhabited Keys on which he introduced a large propagule [500 adult specimens] of *C*. *casablancae* (Fig 1E) in June, 1912. Descendants of these introductions survive little changed on Indian Key after more than a century (Fig 1F). The Bahia Honda population of *C*. *casablancae* remained isolated for more than a decade, but on returning to study this colony in 1931, Bartsch [30] reported the appearance of *C*. *incanum* at the site, as well as the presence of *C*. *incanum x C*. *casablancae* hybrids that persist to the present day. Bartsch [14, 29, 45] monitored the populations he introduced for decades, documenting instances of *Cerion* colonies being extirpated or decimated by fires and hurricanes and of propagules being transported among the Keys by hurricanes.

Our efforts to apportion the specimens in our study to subspecies on the basis of shell characters used to define them met with limited success, as a significant proportion of the shells had phenotypes that were intermediate between those of the named subspecies. With the exception of the nominotypical subspecies *Cerion incanum incanum*, phenotypes corresponding to the type specimens were either entirely absent or a minor component of the populations sampled from their type localities and frequently had broad and discontinuous distributions (Table 2) rather than representing geographically isolated races that are usually associated with subspecies. In his monographic treatment of the terrestrial gastropods of North America, Pilsbry [46], who earlier named two of the subspecies of *Cerion incanum*, treated all of the subspecies as forms, noting that "they have little significance", and listed them as synonyms of *Cerion incanum*.

When COI and 16S sequences were evaluated using the Automatic Barcode Gap Discovery (ABGD) method, all Florida Keys individuals (except for *C*. *casablancae* from Indian Key and individuals from the hybrid population on Bahia Honda Key with mitochondrial genomes inherited from *C*. *casablancae*) were grouped together in the third and all subsequent partitions, indicating the absence of discernible gaps in the distribution of pairwise differences within our sample.

Phylogenetic analyses of the concatenated (16S + COI) sequences affirmed the previously reported derived relationship of *Cerion incanum* to cerionid outgroups, and particularly its sister group relationship to species from the Great Bahama Bank, adding further evidence for a Bahamian origin for the *Cerion* fauna of southeastern Florida and the Florida Keys [26]. The highly supported clade containing all samples of *Cerion incanum*, including all hybrids with mitochondrial genomes derived from *C*. *incanum*, was partitioned into five major groups that formed a ladder-like arrangement (Figs 4 and 5). With the exception of a single specimen from No Name Key, the most basal of these five groups (Clade 1) was composed only of specimens from the older Key Largo Formation (Key Biscayne to Bahia Honda Key). The intermediate clades (Clades 2–4) consisted of specimens that ranged from Plantation Key, the southwesternmost of the High Coral Keys, to Sugarloaf Key, in the central Oölite Keys, while the most derived clade (Clade 5) contained only specimens from the Oölite Keys of the younger Miami Formation (Fig 5). The composition and relationships among the clades of *C*. *incanum* in this phylogeny indicate that the northernmost of the keys, closest to the Tomeu paleoislands that had been inhabited by *Cerion petuchi*, the presumed ancestor of *C*. *incanum*, have the oldest populations, and that this species expanded in a southwesterly direction, colonizing newly emergent Keys as they were formed, with the populations of the Oölite Keys being the youngest.

Phylogenetic analyses as well as haplotype distribution and networks indicate that the faunas of the northern High Coral Keys and the Oölite Keys comprise well supported clades with distinctive haplotypes within each region that are directly interconnected. In contrast, the faunas of the intervening Low Coral Keys consist of heterogeneous groups that include some haplotypes derived from High Coral Keys and others from Oölite Keys. Key Largo of the High Coral Keys and many of the Oölite Keys (eg., Big Pine Key, Sugaloaf Key) are comparatively large islands, each with multiple discontinuous populations of *Cerion*, while Plantation Key (High Coral Keys) and the Low Coral Keys include many small islands, some with a single population of *Cerion*, others without living colonies of *Cerion*.

Upon reaching the Oölite Keys, *Cerion incanum* would have greatly expanded its range across the large land areas cumulatively provided by these Keys. These are topographically low islands, many separated by shallow, narrow channels whose positions and courses could be altered by hurricanes. Stochastic events such as fires and hurricanes, as well as sea level changes during the Holocene would have amplified genetic diversity in *Cerion incanum* inhabiting the Oölite Keys by recombining and isolating the numerous, geographically proximal populations, resulting in the high number of haplotypes occurring on this group of islands [Table 1]. Effects of such stochastic events would be more extreme on *Cerion* populations inhabiting smaller, more isolated Keys separated by greater distances and deeper channels (including, but not limited to the Low Coral Keys). Negative effects could include local extinctions or decimations resulting in genetic bottlenecks, while the likelihood of introductions of new propagules would be inversely proportional to the distance of a Key to a neighboring *Cerion* population. The larger populations of the Oölite Keys and Key Largo were likely more stable over time and served as a source of genetic material to reestablish colonies in the Low Coral Keys or periodically contribute haplotypes to existing small populations.

Discordant patterns in distribution among two mitochondrial genes conflict with the general model that mitochondrial genomes are uniparentally transmitted, homoplastic and nonrecombining, yet similar patterns have been reported in the *Cerion uva* complex in the southernwestern Caribbean [47]. Explanations for this phenomenon range from convergent sequence evolution in haplotypes with broad geographic ranges to heteroplasmy and recombination within mitochondrial genomes. More recent studies have shown that the mitochondrial genome is not always clonal, neutrally evolving or clock-like [48, 49], and have brought to light examples of departures from the uniparental, homoplastic and non-recombining transmission especially in interspecific or interstrain hybrids [50, 51].

In addition to these natural processes by which *Cerion* is transported among islands, railroad bridges connecting Miami to Key West were built between 1905 and 1912, while the Overseas Highway extending to Key West was completed in 1938. Quarries on Windley Key and later on Plantation Key were sources for both fill dirt and slab rock used to build these structures, raising the possibility of more recent anthropogenic transport of *Cerion* propagules in the direction of Key West, perhaps accounting for the anomalous distributions of COI Haplotype 30 and 16S Haplotypes 20 and 26.

### The hybrid population on Bahia Honda Key

Woodruff and Gould [11] provided a succinct summary of the history of Bartsch's introduction of *C*. *casablancae* onto Bahia Honda Key in 1912, including the reduction of the population size to about 55 individuals following a fire in 1915, and the subsequent appearance of *C*. *incanum* at the site by 1931 that resulted in the *C*. *incanum* x *C*. *casablancae* hybrid population present along the southeastern coast of this Key. In a detailed study, these authors analyzed samples of *C*. *incanum*, *C*. *casablancae*, and hybrid populations using multivariate morphometric techniques as well as studies of variation in 17 allozyme loci. They concluded that the hybrids are gradually approaching *C*. *incanum* in shell morphology based on comparisons of samples collected in 1933 and 1977. The distribution of allozymes, which are coded by nuclear genes and subject to Mendelian inheritance, indicated that by 1977 no pure *C*. *casablancae* remained on Bahia Honda and that this introduced genome was diminished by 30% or more in the various localities they sampled. With multiple caveats, they projected that "we may be unable to detect *C*. *casablancae* genes on Bahia Honda Key 350 years from now." [11].

Our sample from southeastern Bahia Honda Key was collected on the coast, south of Woodruff and Gould's station 551, an area that in 1977 had individuals with, on average, only 28% of the *C*. *casablancae* nuclear genome [11]. Our analyses, however, deal only with two mitochondrial genes, which are maternally inherited in species that are panmictic, outcrossing and hermaphroditic. Of the 30 individuals analyzed, 19 (63%) had mitochondrial genomes derived from *C*. *incanum*, and 11 (37%) from *C*. *casablancae*. Changes in the proportions of *C*. *incanum* vs. *C*. *casablancae* mitogenomes in this population over time would require assortive mating, differential mortality or immigration of individuals, or that these mitogenomes have different effects on fecundity, survivorship or generation time. Although we did not conduct multivariate morphometric analyses of shell form, there did not appear to be a conspicuous correlation between shell morphology and mitochondrial lineage.

Eight COI haplotypes and six 16S haplotypes were present among the 19 individuals with the *C*. *incanum* mitogenome. All of the COI haplotypes in this group were endemic to BHSE and within 3 mutational steps of populations on MK, NNK, PK or a haplotype ranging from PK to BTN. Ten individuals were within 4 mutational steps of the population on the southwestern coast of Bahia Honda. In contrast, most of the 16S haplotypes were shared with populations from MK, NNK, BPN or a haplotype ranging from PK to SKN. Endemic haplotypes were within 2 steps of populations on MK, WK or a haplotype present on BPN and LK. Eight specimens were within 2 steps of BHSW. Thus, there is no clear indication as to the geographic origin of the *C*. *incanum* that Bartsch discovered at BHSE in 1931. There were six COI and six 16S haplotypes among the 11 individuals with the *C*. *casablancae* mitogenome. One of these hybrids shared a COI haplotype, and two shared a 16S haplotype with specimens of *C*. *casablancae* from Indian Key. The remaining COI haplotypes differed from those of *C*. *casablancae* by 1 to more than 11 bases, while the remaining 16S haplotypes differed by 1 to 6 bases.

Differences between hybrids with the *C*. *casablancae* mitogenome and *C*. *casablancae* are greater than those between hybrids with the *C*. *incanum* mitogenome and *C*. *incanum*.

The vast majority of proteins present in the mitochondria are encoded by nuclear genes, synthesized in the cytoplasm and imported into the mitochondria. Cytochrome c oxidase is a large, transmembrane protein composed of multiple subunits, of which 11 are nuclear in origin and 3 are encoded by the mitochondrial genome. Although both ribosomal RNA subunits are encoded by mitochondrial genes, there are more than 50 protein components for mitochondrial ribosomes. Proteins coded by nuclear genes are subject to Mendelian inheritance, while proteins coded by mitochondrial genes are generally maternally inherited. In cases of hybridization, subunits derived from the mitochondrial genome of one parent species must combine with subunits encoded by nuclear genes of both parent species in order to produce functional composite molecules, potentially accelerating the evolution of novel variants of mitochondrial genes by processes similar to those that result in hybrizymes [10, 51]. As calculated by Woodruff and Gould [11], the proportion of *C*. *casablancae* genes in the nuclear genome in the hybrid population closest to our sample was 28% in 1977, and was projected to reach zero in less than 350 years. Thus, hybrids with mitogenomes inherited from *C*. *casablancae* have more than twice the number of heterologous nuclear proteins to incorporate into functional multisubunit molecules within the mitochondria than do hybrids with *C*. *incanum* mitogenomes, with the proportion increasing over time. As a consequence, surviving hybrids with *C*. *casablancae* mitogenomes would be expected to exhibit haplotypes with a greater and increasing number of sequence differences than would hybrids with *C*. *incanum* mitogenomes. The proportion of individuals with *C*. *casablancae* derived mitogenomes within hybrid populations may also decrease over time due to selection against reduced mitochondrial function brought about by an increasing load of heterologous protein subunits. The data from our study support both these projections.

Although such large scale transplantation experiments as conducted by Bartsch over a century ago would not be permitted today, the results of his experiments, surviving in the eastern portion of Bahia Honda Key for more than 30 generations, provide an invaluable resource for studies of the long term effects hybridization and especially the interactions of the nuclear and mitochondrial genomes.

## Appendix

Correlation between sequentially numbered haplotypes (bold) in the network analyses in Fig 4 [COI (1–110 + C1–C4)] and Fig 5 [16S (1–75 + C1–C4)] and alphanumeric specimen designations (italics) used in Bayesian analyses in Fig 3. GenBank accession number in square brackets follows the haplotype number.

COI Haplotypes: *Cerion incanum*: **1**[KR080581] (n = 41) = *KB 2*, *4*, *6*, *7*, *10*, *11*, *13*, *15*, *20*, *23*, *29*, *32*, *37*, *38*, *42*, *45* + *KL 1*, *3*, *4*, *5*, *6*, *7*, *8*, *11*, *12*, *13*, *15*, *18*, *19*, *22*, *24* + *KLN 3*, *8*, *10*, *18*, *22*, *24*, *25*, *29*, *30*, *32*; **2**[KR080582] (n = 4) = *KB 8*, *36*,*40*, *53*; **3**[KR080583] (n = 1) = *KB 26*; **4**[KR080584] (n = 1) = *KL 23*; **5**[KR080592] (n = 10) *PK 1*, *7*, *16*, *17*, *20*, *21*, *27*, *29*, *31*, *32*; **6**[KR080593] (n = 1) = *PK 5*; **7**[KR080594] (n = 2) = *PK 10*, *15*; **8**[KR080595] (n = 7) = *KL 2*, *9*, *10*, *14*, *17*, *20*, *21*; **9**[KR080596] (n = 6) = *PK 11*, *19*, *24*, *26*, *28*, *30*; **10**[KR080597] (n = 18) = *KLN 2*, *4*, *5*, *9*, *11*, *12*, *13*, *14*, *16*, *17*, *19*, *20*, *21*, *23*, *26*, *27*, *28*, *31*; **11**[KR080589] (n = 6) = *BHSE 6*, *10*, *14*, *19*, *20*, *32*; **12**[KR080591] (n = 1) = *BHSE 28*; **13**[KR080590] (n = 1) = *BHSE 22*; **14**[KR080585] (n = 1) = *CK 2*; **15**[KR080588] (n = 1) = *NNK 30*; **16**[KR080586] (n = 12) = *MK 14*, *17*, *19*, *21*, *22*, *23*, *26*, *28*, *30*, *36*, *37*, *38*; 1**7**[KR080587] (n = 10) = *MK 1*, *10*, *11*, *13*, *25*, *27*, *32*, *33*, *34*, *35*; **18**[KR080648] (n = 7) = *CK 14*, *24*, *30 + WK 17*, *22*, *28*, *32*; **19**[KR080649] (n = 7) = *WK 4*, *5*, *13*,*19*, *24*, *25*, *27*; **20**[KR080650] (n = 6) = *WK 2*, *18*, *20*, *23*, *30*, *31*; **21**[KR080651] (n = 1) = *WK 26*; **22**[KR080652] (n = 1) = *WK 21*; **23**[KR080653] (n = 5) = *CK 12*, *18*, *25*, *27*, *32*; **24**[KR080654] (n = 6) = *CK 11*, *20*, *21*, *22*, *28*, *29*; **75**[KR080655] (n = 15) = *CK 3*, *4*, *5*, *6*, *7*, *8*, *9*, *10*, *13*, *15*, *17*, *19*, *23*, *26*, *31*; **26**[KR080685] (n = 15) = *NNK 1*, *3*, *4*, *5*, *11*, *12*, *13*, *14*, *16*, *18*, *23*, *25*, *27*, *31*, *32*; **27**[KR080598] (n = 7) = *LK 11*, *15*, *21*, *25*, *28*, *29* + *WK 29*; **28**[KR080599] (n = 6) = *WK 3*, *7*, *10*, *11*, *12*, *14*; **29**[KR080600] (n = 1) = *BPSW 26*; **30**[KR080601] (n = 24) = *BPN 15*, *18*, *22*, *31* + *BPSW 6*, *19*, *21*, *22*, *23*, *24* + *BTN 11*, *30* + *NNK 19*, *28* + *PK 2*, *3*, *4*, *8*, *12*, *13*, *14*, *18*, *22*, *25*; **31**[KR080602] (n = 1) = *PK 6*; **32**[KR080603] (n = 1) = *BHSE 26*; **33**[KR080604] (n = 1) = *BPN 27*; **34**[KR080605] (n = 3) = *NNK 6*, *17*, *24*; **35**[KR080606] (n = 2) = *SKN 18*, *24*; **36**[KR080607] (n = 1) = *SKN 31*; **37**[KR080608] (n = 29) = *BHSW 2*, *4*, *5*, *6*, *7*, *8*, *9*, *10*, *11*, *12*, *13*, *14*, *15*, *17*, *18*, *19*, *20*, *21*, *22*, *23*, *24*, *25*, *26*, *27*, *28*, *29*, *30*, *31*, *32*; **38**[KR080609] (n = 2) = *BHSE 23*, *29*; **39**[KR080610] (n = 1) = *BPN 3*; **40**[KR080611] (n = 4) = *BHSE 11*, *13*, *25*, *27*; **41**[KR080612] (n = 1) = *BHSE 5*; **42**[KR080613] (n = 1) = *BPN 29*; **43**[KR080614] (n = 5) = *BPSW 2*, *8*, *18*, *27*, *30*; **44**[KR080615] (n = 3) = *BPSW 9*, *10*, *32*; **45**[KR080616] (n = 2) = *NNK 8*, *22*; **46**[KR080617] (n = 1) = *BTN 31*; **47**[KR080618] (n = 10) = *BTN 3*, *5*, *7*, *12*, *14*, *21*, *24*, *25*, *27*, *32*; **48**[KR080619] (n = 1) = *BPN 11*; **49**[KR080620] (n = 1) = *BPN 17*; **50**[KR080621] (n = 1) = *BTN 4*; **51**[KR080623] (n = 1) = *BTN 22*; **52**[KR080624] (n = 1) = BTN 26; **53**[KR080622] (n = 1) = *SKN 2*; **54**[KR080625] (n = 3) = *BHSE 2*, *9*, *21*; **55**[KR080626] (n = 3) = *BTN 8*, *10*, *23*; **56**[KR080627] (n = 9) = *BTN 6*, *9*, *13*, *15*,*17*, *20 29 + SKN 14*, *29*; **57**[KR080629] (n = 1) = *SKN 20*; **58**[KR080630] (n = 1) = *SKN 28*; **59**[KR080631] (n = 1) = *SKN 32*; **60**[KR080632] (n = 1) = *SK 4*; **61**[KR080633] (n = 1) = *BTN 2*; **62**[KR080634] (n = 2) = *BTN 16*, *28*; **63**[KR080635] (n = 1) = *BPSW 11*; **64**[KR080636] (n = 2) = *BTN 18*, *19*; **65**[KR080637] (n = 8) = *BPSW 4*, *7*, *12*, *13*, *15*, *16*, *17*, *20*; **66**[KR080638] (n = 1) = *BPSW 14*; **67**[KR080639] (n = 1) = *BPSW 28*; **68**[KR080641] (n = 1) = *LK 5*; **69**[KR080642] (n = 1) = *BPN 10*; **70**[KR080640] (n = 3) = *BPSW 3*, *25*, *31*; **71**[KR080643] (n = 2) = *BPN 4*, *23*; **72**[KR080644] (n = 1) = *BPN 20*; **73**[KR080645] (n = 6) = *BPN 8*, *12*, *13*, *14*, *21*, *25*; **74**[KR080646] (n = 3) = *BPN 19*, *30*, *32*; **75**[KR080647] (n = 2) = *BPN 5*, *6*; **76**[KR080628] (n = 22) = *LK 2*, *3*, *4*, *6*, *7*, *9*, *10*, *12*, *13*, *14*, *17*, *18*, *19*, *20*, *22*, *23*, *24*, *26*, *27*, *30*, *31*, *32*; **77**[KR080678] (n = 1) = *PK 23*; **78**[KR080677] (n = 2) = *WK 6*, *15*; **79**[KR080671] (n = 2) = *KW 12* + *SKN 16*; **80**[KR080679] (n = 1) = *KW 14*; **81**[KR080680] (n = 11) = *SKN 1*, *3*, *4*, *5*, *7*, *9*, *10*, *11*, *12*, *13*, *26*; **82**[KR080681] (n = 6) = *SK 2*, *9*, *12*, *23*, *24*, *32*; **83**[KR080682] (n = 1) = *SK 5*; **84**[KR080683] (n = 3) = *SKN 15*, *22*, *27*; **85**[KR080684] (n = 1) = *SKN 30*; **86**[KR080672] (n = 1) = *SK 25*; **87**[KR080673] (n = 3) = *KW 17*, *20*, *21*; **88**[KR080663] (n = 12) = *KW 1*, *2*, *3*, *6*, *9*, *11*, *13*, *18*, *22*, *25*, *30*, *33*; **89**[KR080662] (n = 8) = *BPN 2*, *7*, *9*, *24*, *28 + SKN 6*,*17*, *19*; **90**[KR080674] (n = 2) = *SKN 21*, *25*; **91**[KR080675] (n = 6) = *KW 10*, *16*, *23*, *24*, *26*, *31*; **92**[KR080676] (n = 4) = *KW 5*, *8*, *15*, *32*; **93**[KR080664] (n = 7) = *SK 6*, *7*, *13*, *16*, *17*, *20*, *21*; **94**[KR080665] (n = 2) = *SK 15*, *22*; **95**[KR080666] (n = 1) = *SKN 23*; **96**[KR080660] (n = 2) = *SK 10*, *27*; **97**[KR080661] (n = 1) = *SK 18*; **98**[KR080658] (n = 2) = *SK 3*, *28*; **99**[KR080659] (n = 1) = *SK 14*; **100**[KR080657] (n = 2) = *SK 11*, *19*; **101**[KR080656] (n = 1) = *SK 26*; **102**[KR080667] (n = 6) = *NNK 2*, *7*, *9*, *15*, *21*, *26*; **103**[KR080668] (n = 3) = *NNK 10*, *20*, *29*; **104**[KR080669] (n = 2) = *BPSW 5*, *29*; **105**[KR080670] (n = 1) = *BPN 26*.

Hybrids: *C*. *incanum* X *C*. *casablancae*: **106**[KR080686] (n = 1) = *BHSE 18*; **107**[KR080687] (n = 2) = *BHSE 24*, *31*; **108**[KR080688] (n = 5) = *BHSE 3*, *8*, *12*, *17*, *30*; **109**[KR080689] (n = 1) = *BHSE 4*; **110**[KR080690] (n = 1) = *BHSE 7*.

*Cerion casablancae*: **C1**[KR080691] (n = 1) = *IK J*; **C2**[KR080692] (n = 2) = *IK F*, *G*; **C3**[KR080693] (n = 2) = *IK E*, *L*; **C4**[KR080694] (n = 2) = *IK C* + *BHSE 15*.

16S Haplotypes: *Cerion incanum*: **1**[KP942955] (n = 25) = *KL 2*, *9*, *10*, *14*, *17*, *20*, *21* + *KLN 2*, *4*, *5*, *9*, *12*, *13*, *14*, *16*, *17*, *19*, *20*, *21*, *23*, *26*, *27*, *28*, *31* + *PK 5*; **2**[KP942956] (n = 29) = *BHSE 6*, *10*, *14*, *20*, *22*, *28*, *32* + *MK 1*, *10*, *11*, *13*, *14*, *17*, *19*, *21*, *22*, *23*, *25*, *26*, *27*, *28*, *30*, *32*, *33*, *34*, *35*, *36*, *37*, *38*; **3**[KP942957] (n = 1) = *KW 5*; **4**[KP942958] (n = 1) = *CK 2*; **5**[KP942959] (n = 1) = *BHSE 19*; **6**[KP942960] (n = 1) = *NNK 30*; **7**[KP942961] (n = 5) = *PK 11*, *19*, *26*, *28*, *30*; **8**[KP942962] (n = 1) = *PK 24*; **9**[KP942963] (n = 4) = *PK 27*, *29*, *31*, *32*; **10**[KP942964] (n = 3) = *PK 16*, *17*, *20*; **11**[KP942965] (n = 5) = *PK 1*, *7*, *10*, *15*, *21*; **12**[KP942966] (n = 1) = *KLN 11*; **13**[KP942967] (n = 1) = *KB 15*; **14**[KP942968] (n = 7) = *KB 6*, *7*, *10*, *23*, *29*, *38*, *45*; **15**[KP942969] (n = 22) = *KB 2*, *4*, *8*, *11*, *13*, *20*, *32*, *36*, *37*, *40*, *42*, *53* + *KLN 3*, *8*, *10*, *18*, *22*, *24*, *25*, *29*, *30*, *32*; **16**[KP942970] (n = 16) = *KL 1*, *3*, *4*, *5*, *6*, *7*, *8*, *11*, *12*, *13*, *15*, *18*, *19*, *22*, *23*, *24*; **17**[KP942971] (n = 1) = *KB 26*; **18**[KP942972] (n = 2) = *SKN 5*, *10*; **19**[KP942973] (n = 3) = *NNK 10*, *20*, *29*; **20**[KP942974] (n = 75) = *BPN 7*, *26*, *28* + *BPSW 5*, *29* + *KW 1*, *2*, *3*, *6*, *8*, *9*, *10*, *11*, *13*, *14*, *15*, *16*, *17*, *18*, *20*, *21*, *22*, *23*, *24*, *25*, *26*, *30*, *31*, *32*, *33* + *LK 2*, *3*, *4*, *6*, *7*, *9*, *10*, *12*, *13*, *14*, *17*, *18*, *19*, *20*, *22*, *23*, *24*, *26*, *27*, *30*, *31*, *32 + PK 23 + SK 2*, *4*, *5*, *9*, *10*, *12*, *18*, *23*, *24*, *27*, *32* + *SKN 4*, *9*, *12*, *15*, *21*, *22*, *23*, *25*, *27* + *WK 6*, *15*; **21**[KP942975] (n = 7) = *BPN 2 + NNK 2*, *7*, *9*, *15*, *21*, *26*; **22**[KP942976] (n = 6) = *SK 3*, *11*, *14*, *19*, *26*, *28*; **23**[KP942977] (n = 1) = *SKN 3*; **24**[KP942978] (n = 1) = *SKN 6*; **25**[KP942979] (n = 1) = *SKN 16*; **26**[KP942980] (n = 52) = *BHSE 26* + *BPN 5*, *6*, *10*, *15*, *17*, *18*, *20*, *22*, *27*, *29*, *31* + *BPSW 4*, *7*, *11*, *12*, *13*, *15*, *16*, *17*, *20*, *25*, *26*, *28*, *31* + *BTN 3*, *4*, *5*, *6*, *7*, *9*, *10*, *11*, *12*, *13*, *14*, *21*, *23*, *24*, *25*, *27*, *30*, *31*, *32* + *PK 4*, *8*, *12*, *13*, *14* + *SKN 2*, *20*, *28*; **27**[KP942991] (n = 7) = *CK 28 + LK 11*, *15*, *21*, *25*, *28*, *29*; **28**[KP942990] (n = 1) = *SK 25*; **29**[KP942988] (n = 2) = *SK 15*, *22*; **30**[KP942989] (n = 2) = *BTN 18*, *19*; **31**[KP942992] (n = 1) = *BPN 11*; **32**[KP942993] (n = 4) = *SKN 1*, *7*, *26*, *30*; **33**[KP942994] (n = 1) = *SKN 13*; **34**[KP942995] (n = 1) = *SKN 11*; **35**[KP942997] (n = 1) = *PK 6*; **36**[KP942996] (n = 15) = *NNK 1*, *3*, *4*, *5*, *11*, *12*, *13*, *14*, *16*, *18*, *23*, *25*, *27*, *31*, *32*; **37**[KP943001] (n = 28) = *BHSW 2*, *4*, *5*, *6*, *7*, *9*, *10*, *11*, *12*, *13*, *14*, *15*, *17*, *18*, *19*, *20*, *21*, *22*, *23*, *24*, *25*, *26*, *27*, *28*, *29*, *30*, *31*, *32*; **38**[KP943002] (n = 1) = *BHSW 8*; **39**[KP943000] (n = 3) = *BHSE 23*, *29+ NNK 19*; **40**[KP943007] (n = 5) = *BTN 2*, *16*, *17*, *28*, *29*; **41**[KP943004] (n = 6) = *BPSW 6*, *19*, *21*, *22*, *23*, *24*; **42**[KP943005] (n = 7) = *BPSW 2*, *8*, *10*, *18*, *27*, *30*, *32*; **43**[KP943006] (n = 1) = *BPSW 9*; **44**[KP943003] (n = 2) = *BPN 3* + *BPSW 3*; **45**[KP943008] (n = 2) = *BPN 4*, *23*; **46**[KP943009] (n = 1) = *BTN 8*; **47**[KP943010] (n = 5) = *BTN 15*, *20 + NNK 6*, *17*, *24*; **48**[KP943011] (n = 3) = *NNK 8*, *22*, *28*; **49**[KP943012] (n = 2) = *SKN 14*, *29*; **50**[KP943013] (n = 1) = *SKN 32*; **51**[KP943019] (n = 1) = *BPSW 14*; **52**[KP943020] (n = 1) = *BTN 26*; **53**[KP943021] (n = 1) = *BTN 22*; **54**[KP943018] (n = 8) = *BHSE 5*, *11*, *13*, *25*, *27* + *BPN 19*, *30*, *32*; **55**[KP943022] (n = 1) = *KW 12*; **56** [KP943024] (n = 2) = *SKN 18*, *24*; **57**[KP943025] (n = 1) = *SKN 31*; **58**[KP943017] (n = 6) = *WK 7*, *10*, *11*, *12*, *14*, *29*; **59**[KP943023] (n = 1) = *WK 3*; **60**[KP943014] (n = 2) = *PK 18*, *25*; **61**[KP943015] (n = 3) = *PK 2*, *3*, *22*; **62**[KP943016] (n = 3) = *BHSE 2*, *9*, *21*; **63**[KP942987] (n = 7) = *BPN 8*, *12*, *13*, *14*, *21*, *25 + LK 5*; **64**[KP942981] (n = 2) = *SKN 17*, *19;* **65**[KP942998] (n = 1) = *BPN 24*; **66**[KP942999] (n = 1) = *BPN 9;* **67**[KP942982] (n = 7) = SK 6, 7, 13, 16, 17, 20, 21; **68**[KP942983] (n = 24) = *CK 11*, *20*, *21*, *22*, *29* + *WK 2*, *4*, *5*, *13*, *17*, *18*, *19*, *20*, *21*, *22*, *23*, *24*, *25*, *26*, *27*, *28*, *30*, *31*, *32*; **69**[KP942986] (n = 3) = *CK 14*, *24*, *30*; **70**[KP942984] (n = 5) = *CK 12*, *18*, *25*, *27*, *32*; **71**[KP942985] (n = 15) = *CK 3*, *4*, *5*, *6*, *7*, *8*, *9*, *10*, *13*, *15*, *17*, *19*, *23*, *26*, *31*.

Hybrids: *C*. *casablancae* X *C*. *incanum*: **72**[KP943029] (n = 6) = *BHSE 3*, *12*, *17*, *24*, *30*, *31*; **73**[KP943030] (n = 1) = *BHSE 4*; **74**[KP943031] (n = 1) = *BHSE 8*; **75**[KP943032] (n = 1) = *BHSE 18*.

*Cerion casablancae*: **C1**[KP943026] (n = 4) = *IK C*, *F*, *G* + *BHSE 15*; **C2**[KP943027] (n = 2) = *IK E*, *L*; **C3**[KP943028] (n = 2) = *IK J + BHSE 7*.
